# Draft genome of *Metabacillus niabensis* strain 4T19^T^ isolated from cotton-waste composts for mushroom cultivation

**DOI:** 10.1016/j.nmni.2021.100894

**Published:** 2021-05-08

**Authors:** L.J. Kangale, A. Levasseur, D. Raoult, E. Ghigo, P.-E. Fournier

**Affiliations:** 1) Aix-Marseille Univ, IRD, AP-HM, SSA, VITROME, Marseille, France; 2) IHU-Méditerranée-Infection, Marseille, France; 3) Aix-Marseille Univ, IRD, AP-HM, MEPHI, Marseille, France; 4) Special Infectious Agents Unit, King Fahd Medical Research Center, King Abdulaziz University, Jeddah, Saudi Arabia; 5) Techno-Jouvence, 19-21 Boulevard Jean Moulin, 13385, Marseille cedex 05, France

**Keywords:** Cotton-waste, genome, *Metabacillus niabensis* strain 4T19^T^, mushroom, planarian, CSUR, Collection de Souches de l’Unitédes Rickettsies., DSMZ, German Collection of Microorganisms and Cell Cultures

## Abstract

In this article, we present the draft genome sequence of *Metabacillus niabensis* strain 4T19^T^ (= CSUR Q2603 ^T^ = DSM 17723 = JCM 16399 = KACC 11279), that is a new *Metabacillus* species isolated from cotton-waste composts**.** The genome sequence from *Metabacillus niabensis* strain 4T19^T^ was assembled into 462 contigs for a total size of 4,987,608 bp with a G + C content of 35.5%.

## Announcement

*Metabacillus fastidiosus* is considered as type species of genus *Metabacillus* [1]. Strain 4T19^T^ was isolated from cotton-waste composts for mushroom cultivation and was described as type strain of *Metabacillus niabensis* [2]. Working on planarians microbiota, we identified a bacterial strain, Marseille-P9898 (= CSUR P9898 = DSM 111480). We found that Marseille-P9898 strain had similarity at the level of a 16S rRNA gene sequence with *Metabacillus niabensis* strain 4T19^T^ (98.99%). This 16S rRNA gene similarity value does not discriminate the strain Marseille-P9898 from the strain 4T19^T^. However, the characterisation of the strain Marseille-P9898 by a taxonogenomics approach requires the genome from the strain 4T19^T^, but to date, no genome sequence is available for the strain 4T19^T^. Thus, here, we describe the draft genome of *Metabacillus niabensis* strain 4T19^T^. We purchased the strain 4T19^T^ (=DSM 17723) from the Deutsche Sammlung von Mikroorganismen und Zellkulturen GmbH and deposited in the Collection de Souches de l’Unité des Rickettsies (CSUR) culture collection under the number Q2603. Strain 4T19^T^ was grown at 28°C for 24 hours on Columbia agar supplemented with 5% sheep blood (bioMérieux, Marcy l’Etoile, France) in aerobic atmosphere. To extract bacterial genomic DNA (gDNA) of the strain Q2603, a mechanical treatment was first performed on a single colony, by glass beads acid washed (Sigma Aldrich Chimie, Saint-Quentin-Fallavier, France) using a FastPrep-24™ 5G Grinder (MP Biomedicals, Illkirch, France) at maximum speed (6.5) for 90 s. Then after a 30-minute lysozyme incubation at 37°C, DNA (gDNA) was extracted using an EZ1 BioRobot and the EZ1 DNA tissue kit (Cat No./ID: 953034, Qiagen, Hilden, Germany). The gDNA was sequenced using MiSeq technology (Illumina Inc, San Diego, CA, USA) [3] with the Nextera XT DNA sample prep kit (Illumina), and mate-pair strategy. Foremost, gDNA was fragmented, then amplified to 12 cycle of PCR, followed by the addition of the tag adapters and introduced dual-index barcodes. Libraries were purified on AMPure XP beads (Beckman Coulter Inc, Fullerton, CA, USA), then normalised on specific beads following the Nextera XT protocol and pooled into a single library for sequencing. After 39 hours, automated cluster generation and paired-end sequencing with dual-index reads were performed in a single run in 2 × 250-bp format. The 6,897,812 paired-end reads of the Miseq run were checked in accordance with quality using FastQC 0.11.8 [9] and trimmed using Trimmomatic 0.36.6 [8], with default parameters. Trimmed reads were assembled using the Spades [4] genome assembler software (Galaxy 3.12.0+galaxy1) set with default parameters. Default parameters were applied here and for all software (for k values, i.e. k-mer values of 127, 99, 77, 55, 33 and 21). Using default parameters of SSAKE-based Scaffolding of Pre-Assembled Contigs after Extension (SSPACE 2.0) [5] and GapFiller (GapFiller 1.10) [6] allowed us to combine the contigs. Briefly, we have obtained 792 scaffolds after assembly, then using SSAKE, we reduced to 520 scaffolds and the manual finishing allowed us to obtain 462 scaffolds (singletons = 473, multicontig scaffold = 15), then manual finishing by using sequence similarity searches and syntax block detection between closest species in the *Metabacillus* genus (using type strain of the *Metabacillus halosaccharovorans, Metabacillus litoralis* and *Metabacillus fastidiosus*). For contigs profiling, a BLAST e-value threshold of 1e-10 was set to perform similarity searches and contig length threshold was set to 500 pb and a contig coverage threshold of 15% was applied. Finally, *Metabacillus niabensis* strain 4T19^T^ was assembled into 462 contigs (N_50,_ 19,230 bp; L_50,_ 64; coverage, 5x) for a total size of 4,987,608 bp, with a G + C content of 35.5%. Genomic annotation was obtained by NCBI prokaryotic genome annotation pipeline (pGAP) [7]. A total of 4969 genes were identified, along with 11 rRNAs, 61 tRNAs, 6 ncRNA and 1tmRNA. A genomic comparison considering the size, GC% and contig was performed (Table 1), evidencing that *Metabacillus niabensis* is different of the other species of *Metabacillus*. To note, *Metabacillus niabensis* is a motile Gram negative and aerobic bacteria, growing from 15 to 40°C. *Metabacillus niabensis* is catalase and betagalactosidase positive, and oxidase negative, and the principal fatty acid found in this bacterium is the 12-methyl-tetradecanoic acid [10].

**Table 1:** Genomic comparison of *Metabacillus niabensis* with others genus *Bacillus, Mesobacillus, Metabacillus* and *Neobacillus*

Name	Size (bp)	GC%	Contigs	Refseq
*Bacillus flexus*	3,906,163	37.6	259	BCVD01000001.1
*Bacillus acidicola*	5,137,992	39.4	10	LWJG01000001.1
*Mesobacillus foraminis*	5,730,823	43.0	35	SLVV01000001.1
*Metabacillus halosaccharovorans*	5,399,327	36.1	8	MTIR01000001.1
*Metabacillus litoralis*	5,230,624	35.9	1	NZ_CP033043.1
*Metabacillus niabensis*	4,987,608	35.5	462	NZ_CADEPK010000000
*Neobacillus niacini*	2,201,253	38.3	143	JRYQ01000001.1
*Metabacillus fastidiosus*	4,410,645	35.1	239	NZ_BCVG00000000.1
*Mesobacillus subterraneus*	4,571,170	43.9	42	RSFW01000001.1
*Bacillus cohnii*	4,899,141	36.1	1	NZ_CP018866.1

Genomic comparison considering the size, GC% and contigs. Refseq: numbers reference sequence in NCBI.

## Data availability

The complete 16S rRNA gene sequence of *Metabacillus niabensis* have been deposited at GenBank under the accession numbers AY998119. The genome sequence *Metabacillus niabensis* have been deposited at GenBank under the accession number CADEPK010000000 (https://www.ncbi.nlm.nih.gov/Traces/wgs/CADEPK01?display = contigs&page = 1). Illumina MiSeq paired-end sequencing raw data have been deposited under accession number ERR4020000 (https://www.ncbi.nlm.nih.gov/sra/ERR4020000). The *Metabacillus niabensis* strain 4T19^T^ is available at the Collection under the reference CSUR Q2603 = DSM 17723 = JCM 16399 = KACC 11279.

## Author contribution statement

LJK conceived the experiments, realised the experiments, analysed the data, prepared figures, wrote the manuscript. DR, EG, AL and PEF designed the experiments, conceived the experiments, analysed the data, wrote the manuscript.

## Conflict of interest

The authors have no conflicts of interest to declare. The funding sources had no role in the study design, data collection and analysis, decision to publish or manuscript preparation.
